# Hormonal Neuroendocrine and Vasoconstrictor Peptide Responses of Ball Game and Cyclic Sport Elite Athletes by Treadmill Test

**DOI:** 10.1371/journal.pone.0144691

**Published:** 2015-12-30

**Authors:** Anna Protzner, Márta Szmodis, Anna Udvardy, Edit Bosnyák, Emese Trájer, Zsolt Komka, István Györe, Miklós Tóth

**Affiliations:** 1 Department of Health Sciences and Sport Medicine, University of Physical Education, Budapest, Hungary; 2 Research Institute of Hungarian Armed Forces, Aeromedical Hospital, Kecskemét, Hungary; Cleveland Clinic, UNITED STATES

## Abstract

**Objective:**

Our objective was to evaluate complex hormonal response in ball game and cyclic sport elite athletes through an incremental treadmill test, since, so far, variables in experimental procedures have often hampered comparisons of data.

**Methods:**

We determined anthropometric data, heart rate, maximal oxygen uptake, workload, plasma levels of lactate, adrenaline, noradrenaline, dopamine, cortisol, angiontensinogen and endothelin in control (n = 6), soccer (n = 8), handball (n = 12), kayaking (n = 9) and triathlon (n = 9) groups based on a Bruce protocol through a maximal exercise type of spiroergometric test.

**Results:**

We obtained significant increases for adrenaline, 2.9- and 3.9-fold by comparing the normalized means for soccer players and kayakers and soccer players and triathletes after/before test, respectively. For noradrenaline, we observed an even stronger, three-time significant difference between each type of ball game and cyclic sport activity.

**Conclusions:**

Exercise related adrenaline and noradrenaline changes were more pronounced than dopamine plasma level changes and revealed an opportunity to differentiate cyclic and ball game activities and control group upon these parameters. Normalization of concentration ratios of the monitored compounds by the corresponding maximal oxygen uptake reflected better the differences in the response level of adrenaline, noradrenaline, dopamine and cortisol.

## Introduction

Stress is a physical and emotional response to environmental stimuli disturbing homeostasis. Physical exercise can be considered as an acute stress. The stress response can be assumed as a neuroendocrine mechanism that occurs in anticipation of physical exercise. Catecholamines play a major role in regulation of oxygen and energetic substrates (i.e., glucose) transportation to active muscles during prolonged exercise [1]. Having consulted the literature in the field there are studies where a decrease in plasma catecholamine concentrations in response to exercise could be observed [2]. Winder et al. reports that the magnitude of the increase in catecholamine levels is less in endurance-trained individuals than in untrained subjects [3]. Some other studies propose conversely, the more intense exercise, plasma noradrenaline concentrations appear to be significantly higher in endurance-trained compared with untrained subjects [4]. It is not clear what effect the training has on the catecholamine response to exercise however high capacity to secrete noradrenaline (NA) and adrenaline (A) may represent an advantage in competitive sports [5].

Research on DA level changes during exercise showed negligible changes in plasma DA detected in young athletes related to short [6] or long [7] duration of physical performance. For DA, a slower release mechanism was suggested than for NA [8].

The role of angiotensinogen (AGT) in the regulation of blood pressure as well as sodium and water homeostasis is well recognized [9].

Endothelin (ET) belongs to a family of potent vasoconstrictor peptides synthesized by vascular endothelial cells having a prolonged vasoconstrictor activity and also a direct arrhythmogenic and inotropic effect. ET concentration may be increased by prolonged strenuous exercise [10, 11]. Recent evidence indicates an emerging pathophysiologic role of ET-1 during myocardial ischaemia and evolving infarction [12].

Cortisol (C) is secreted by the adrenal gland and it regulates most physiological/metabolic adaptations to exercise training. Cortisol response depends on exercise intensity and duration, fitness level, nutritional status and circadian rhythm. Kraemer et al. observed high C level in cyclic sport athletes [13].

Catecholamine concentrations are influenced by several factors in athletes such as exercise characteristics, duration of exercise, training status and gender [14–17]. However, published data are conflicting maybe due to the lack of an approach to evaluate complex hormonal responses. Hormonal responses of endurance athletes has already been in the focus of research interest and it is better described than the sports which involve different category of training that cannot be classified only as endurance or strength training such as handball, soccer, kayaking and triathlon.

The aim of the present study was to determine hormonal neuroendocrine and vasoconstrictor peptide variations in individual and team athletes of cyclic and ball game sport activities, respectively, after maximal exercise by executing the same study design. Due to the serum hormonal and vasoconstrictor peptide levels it was important to standardize the environment of the research, however it is known that a general conclusion from a single type of stress test cannot be made and refer to a field training regimen.

## Methods

### Participants

Forty-four non-smoking, healthy Caucasian Hungarian male subjects of two team (soccer and handball), two individual sports (triathlon and kayaking) corresponding to ball game and cyclic physical activities, respectively, and controls participated on a voluntary basis in the present study. We classified kayaking and triathlon as cyclic sport activities according to Ahmetov et al.[18]. National elite athletes volunteered, while students of the Semmelweis University of Medicine in Budapest were monitored as controls. Sprint-distance (750-meter swim, 20-kilometer bike and 5-kilometer run) triathletes took part in the present study. The flat water K1, K2 and K4 kayakers trained for races of 500 m, 1000 m and 2000 m, respectively. The identification code (ID) of controls, handball, soccer, kayaking and triathlon were 1–6, 7–18, 19–26, 27–35 and 36–44, respectively. Participants performed one maximal exercise test to determine VO_2max_ and maximal workload. Controls practiced only aerobic physical activities on a regular basis with weekly training times ranging less than 3 h.

We conducted the study at the University of Physical Education, Budapest, with athletes being in their preparatory phase of the forthcoming competitions each day between 10 AM and 4 PM.

## Research Ethics Approval

All subjects read and signed an informed-consent form approved by the Public Health Department of the Government Office of Budapest. The protocol was approved by the Semmelweis University’s institutional review board and the Medical Research Council of Hungary by the Scientific and Research Ethics Committee. The experiments carried out in this study comply with the current laws of Hungary.

### Workload protocol

We used an Ergosana ERG 911 treadmill equipped with a Cardiovit AT-104 ECG recorder (Schiller Medizintechnik GmbH, Ottobrun, Germany), in conjunction with a Powercube^®^ O_2_ and carbon-dioxide (CO_2)_ gas analysis unit supplied by Ganshorn (Niederlauer, Germany). We calibrated the gas analyzer after each measurements. Basic criterias to evaluate VO_2max_ were reaching the plateau in oxygen uptake, respiratory exchange ratio (higher than 1.1) and 90% of age predicted HRmax [19]. Tests were terminated if subjects achieved maximal oxygen uptake criteria or reported dizziness or muscle fatigue. Modified Bruce protocol has been applied as a maximal exercise type of spiroergometric test performed on the aforementioned treadmill. Briefly, the modified Bruce protocol was of ascending grade type starting at 0% for all volunteers with a constant running speed of 9 km h^-1^ for 4 min and 6 km h^-1^ for 4 min for the athletes and controls, respectively. Then, increments of 1.5% were applied every minute, with a constant running speed of 12 km h^-1^ and 9 km h^-1^ for athletes and control group, respectively.

### Lactate measurement

We measured lactate (LAC) concentration in a blood drop taken from the ear lobe of each volunteer by using a blood LAC measuring meter supplied by Nova Biomedical (Waltham, Massachusetts, USA). The accuracy of determination of LAC was assured from the spiking of real samples with a standard known concentration. Lactate was determined from the ear lobe of each participant by using a blood lactate measuring meter supplied by Nova Biomedical (Waltham, Massachusetts, USA). Briefly, the sampled blood drop should be touched to the supplied strip. Measuring range for lactate is between 0.3 to 25 mM whole blood. The area of skin of the ear lobe from where blood was collected at the basal and after the achievement of the maximal oxygen uptake criteria (after the test).

### Determination of catecholamines, angiotensinogen, cortisol and endothelin levels

We determined levels of A, NA, DA, AGT, ET and C from plasma by solid phase enzyme-linked immunosorbent assay (ELISA) using a TriCat ELISA kit supplied by IBL—International (Hamburg, Germany) at the R&D laboratory of Diagnosztikum Co. (Budapest, Hungary) From each volunteer, we took 12 mL of blood at the basal and after the maximal workload levels into Vacuette tubes (Santa Cruz Biotechnology Inc., Heidelberg, Germany) coated with K3 EDTA. We subjected samples to centrifugation at 3,000 g and 4°C for 10 minutes and then, we further divided into six aliquots (2 mL each) for the determination of A, NA, DA, AGT, ET and C. In the case of A, NA and DA, we added 200 μL aprotinin 6 mL^-1^ whole blood (Gordox) prior to centrifugation. The accuracy of determination of catecholamines, C, AGT and ET was assured by spiking of real samples with a standard known concentration.

### Statistical analysis

**Fig 1:**
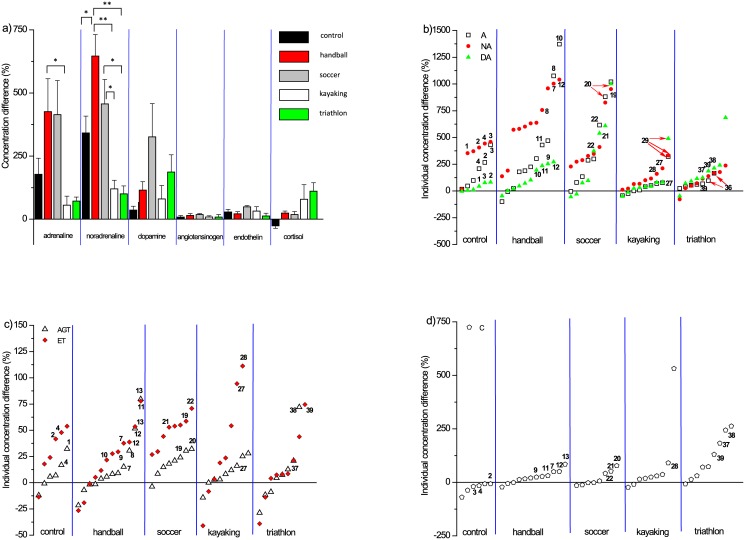
Differences in the mean (± standard error, SE) (a) and individual concentration of catecholamines (b), vasoconstrictor peptides (c) and cortisol (d) between after and before test for the volunteers normalized with the basal concentration level of each corresponding neuroendocrine hormone and vasoconstrictor peptide. Significant differences (*p* < 0.05 and *p* < 0.01) are indicated with one or two asterisk(s), respectively; See text for exact *p* values. Arabic numbers in Fig 1b–1d represent the volunteer identification code in the given group for which the difference in at least three hormone concentration levels after and before test was higher than the mean of the hormonal concentration difference.

All data are expressed as means ± standard deviation (SD) except Fig 1a where ± standard error (SE) was used to solute the representation. Because of the limited sample size to investigate normality, Shapiro-Wilkes normality test was performed. As all data were found to be normal, parametric statistical method was used afterwards. Concerning our results, Student’s paired *t*-test for dependent samples was the adequate statistical method to describe differences gathered after and before the exercise test. ANOVA was the adequate method for comparing differences in data for concentration variables with more than two independent samples. As *post hoc* test, we applied the Tukey honest significant difference method for different sample sizes (Statistica 11.0 software, StatSoft, Tulsa, Oklahoma, USA). We set the significance level at *p* < 0.05 or *p* < 0.01 for all variables.

## Results

### Anthropometric and exercise data

The anthropometric and exercise data of the participants are summarized in Tables 1 and 2, respectively. The average age of the athletes was similar (22.2 ± 4.0 years) except for the members of the handball team members whose age was 26.3 ± 3.5 years, Table 1.

**Table 1:** Anthropometric data of the participants involved in the investigation (mean ± standard deviation, SD)

Sport activity	Volunteer	N° volunteers (n)	Training experience (years)	Age ± SD (years)	Weight ± SD (kg)	Height ± SD (cm)	Fractionated mass^1^			
							fat	muscle	bone	residual
							(% ± SD)	(% ± SD)	(% ± SD)	(% ± SD)
	Control	6	-	23.5 ± 1.9	76.9 ± 10	179.7 ± 2.7	13.9 ± 4.3	44.4 ± 3.3	16.7 ± 1.3	25.0 ± 1.7
Ball games	Handball	12	15.6 ± 3.2	26.3 ± 3.5	101.7 ± 9.5	192.2 ± 6.9	14.4^b^ ± 3.1	44.2 ± 2.2	16.6 ± 1.1	24.8 ± 1.1
	Soccer	8	10.2 ± 1.3	20.8 ± 2.3	79.4 ± 7.2	181.1 ± 7.5	9.02^a^ ± 1.5	48.0 ± 1.0	16.5 ± 0.9	26.5 ± 1.1
Cyclic	Kayaking	9	11.2 ± 0.8	19.5 ± 2.3	85.9 ± 8.1	184.1 ± 6.2	10.2 ± 0.8	47.9 ± 1.2	16.0 ± 0.5	25.9 ± 1.3
	Triathlon	9	10.0 ± 2.1	20.4 ± 2.0	68.6 ± 12.0	177.6 ± 7.6	11.3 ± 2.2	44.3 ± 4.1	16.8 ±0.9	27.5 ± 2.2

^1^ according to Drinkwater and Ross (1980)

^a^ significant difference (*p* = 0.0326) compared to the control group;

^b^ significant difference (*p* = 0.0124) compared to the soccer athletes

**Table 2:** Exercise parameters after the treadmill test (mean values ± SD).

Sport activity	Volunteer	HR_max_	VO_2max_	Relative aerobic capacityVO_2max_ BW^-1^	LAC_max_	Cumulative workload(W)
		(beats min^-1^)	(mL min^-1^)	(mL min^-1^ kg^-1^)	(mmol L^-1^)	
	Control	198 ± 8	3572 ± 438	47.0 ± 7.8	11.0 ± 1.7	2796 ± 885
Ball games	Handball	184 ± 12	4683^1^ ± 563	48.2 ± 9.1	10.7 ± 1.9	3886^2^ ± 591
	Soccer	195 ± 10	4209 ± 616	54.4 ± 5.6	12.4 ± 1.9	3440 ± 593
Cyclic	Kayaking	194 ± 8	4358 ± 652	56.4 ± 4.1	12.0 ± 0.9	3547 ± 313
	Triathlon	198 ± 12	4541^1^ ± 788	66.1^3^± 13.9	12.3 ± 3.6	4690^2^ ^,^ ^a^ ± 887

Abbreviations: HR_max_ = maximal heart rate; LAC_max_ = maximal lactate level; VO_2max_ = maximal oxygen uptake;

^1^ significant difference for handball (*p* = 0.00874) and triathlon (*p* = 0.0496) compared to the control group;

^2^ significant difference for handball (*p* = 0.0249) and triathlon (*p* = 0.000194) compared to the control group,

^3^ significant difference *p* being 0.00628 and 0.00249 compared to the control group and handball athletes, respectively;

^a^ significant difference *p* being 0.00572 and 0.0183 compared to the soccer and kayaking athletes, respectively.

*Maximal lactate level was measured at the end of the exercise

The statistical analysis by ANOVA did not reveal any significant difference in the fractionated mass data of all volunteers except for the body fat percentage between handball and soccer players as well as control group and soccer players (Table 1).

The relative aerobic capacity expressed as VO_2max_ BW^-1^ was significantly higher for triathletes compared to control and handball volunteers, whereas the mean VO_2max_ and cumulative workload of triathletes were also significantly higher compared to the control group and soccer and kayak volunteers (Table 2).

### Intragroup hormonal changes

The minimum, maximum and mean concentration of A, NA, DA, AGT, ET and C in all volunteers expressed as nmol L^-1^ ± SD before and after the treadmill test are listed in Table 3. By looking at the *intra*group hormonal variability, we observed significant differences between the A mean values for the control (*p* = 0.0261), ball game (*p* = 0.0015 and *p* = 0.0148 for handball and soccer athletes, respectively) and triathlon volunteers (*p* = 0.0012). For NA mean values before and after executing the exercise protocol, there was a significant difference for each group (*p* = 0.0074, *p* = 0.0001, *p* = 0.0001 and *p* = 0.0062 for control, handball, soccer and kayaking, respectively) except for triathletes. For ET, we observed significant differences between the maximal and basal values for control (*p* = 0.0291) and ball game players (*p* = 0.0277 and *p* = 0.0001 for handball and soccer volunteers, respectively). Generally, there were few significant changes in the case of DA, C and AGT levels in the case of comparing before and after the test values.

**Table 3:** Concentration (mean ± SD, minimum–maximum range) of adrenaline (A), noradrenaline (NA), dopamine (DA), angiotensinogen (AGT), endothelin (ET) and cortisol (C) in athletes expressed as nmol L^-1^ before and after treadmill test.

Sport activity	Volunteer	A		NA		DA	
		mean ± SD		mean ±SD		mean ±SD	
		min–max		min–max		min–max	
treadmill test		before	after	before	after	before	after
	Control	23.3 ± 8.9	56.3^1^ ± 20.9	172 ± 60	704^2^ ± 291	15.5 ±2.1	21.1 ± 6.2
		14.1–38.6	31.7–93.0	91.4–271	268–1225	11.4–18.1	14.5–33.1
Ball games	Handball	9.9–53.3	136^2^ ± 53	267 ± 88	1719^2^ ± 607	25.7 ± 12.6	43.7 ± 15.3
		40.1 ± 21.7	59.2–229	176–509	618–2465	<0.15–46.0	24.9–81.5
	Soccer	29.1–214	299^2^ ± 169	457 ± 158	2204^2^ ± 413	34.2 ± 26.7	74.6^1^ ± 27.4
		84.8 ± 61.4	153–626	193–637	1551–2843	8.3–83.5	26.9–105
Cyclic	Kayaking	27.6–87.6	76 ± 34	218 ± 79	461^2^ ± 230	27.2 ± 20.2	32.6 ± 17.2
		56.5 ± 21.6	37.2–135	134–362	231–955	4.0–57.2	16.7–68.8
	Triathlon	32.8–82.8	87.7^2^ ± 25.5	234 ± 83	414 ±178	12.3 ± 6.1	27.6^2^ ± 10.6
		57.5 ± 18.6	55.5–127	152–432	90.7–730	4.8–23.1	12.2–43.0
		AGT		ET		C	
		mean ±SD		mean ±SD		mean ±SD	
		min–max		min–max		min–max	
treadmill test		before	after	before	after	before	after
	Control	2.7 ± 0.4	2.9 ± 0.4	6.4 ± 1.3	8.2^1^ ± 1.9	159 ± 38	119 ±50
		2.2–3.5	2.4–3.5	5.1–9.3	5.3–11.5	98.2–216	55.8–200
Ball games	Handball	3.2 ± 0.8	3.6 ± 1.0	7.4 ± 2.6	8.8^1^ ± 3.0	130 ± 45	155^1^ ± 46
		2.0–4.7	2.1–4.6	4.8–15.1	4.2–15.9	83.4–222	104–250
	Soccer	3.0 ± 0.6	3.5^2^ ± 0.6	10.9 ± 1.5	16.1^2^ ± 1.0	141 ± 46	165 ±71
		2.0–3.9	2.4–4.4	9.5–13.1	14.6–17.2	73.7–223	91.8–316
Cyclic	Kayaking	3.1 ± 0.6	3.3 ± 0.8	10.5 ± 2.9	13.8 ± 6.6	164 ± 103	188 ± 99
		2.2–4.0	2.4–4.7	6.2–13.4	6.0–26.0	3.7–333	23.0–381
	Triathlon	3.9 ± 0.9	4.2 ± 1.0	11.8 ± 3.9	12.6 ±3.3	119 ± 51	211^2^ ± 40
		2.5–5.3	2.9–5.5	6.3–16.4	7.6–19.2	51.9–205	163–293

Significant intragroup changes (^1^, if p < 0.05 and ^2^, if p < 0.01). See text for exact p values.

For each group of volunteers, the NA and A concentration ratios after and before the test were always higher than one (Table 4). The ratios of LAC concentration levels after reaching the maximal workload and at basal level were the highest for controls and the lowest for triathletes. The concentration ratios of A and LAC levels determined after reaching the maximal workload were similar for the control group, kayaking and triathlon volunteers but were considerably higher for ball game athletes by a factor of 4.7 and 2.5 for soccer and handball athletes, respectively (Table 4).

**Table 4:** Adrenaline (A), noradrenaline (NA) and lactate (LAC) concentration ratios after and before the applied treadmill test.

Sport activity	Volunteer	LAC_max_/LAC_0_	A_max_/LAC_max_	NA_0_/A_0_	NA_max_/A_max_
	Control	9.2	5.1	7.4	12.5
Ball games	Handball	6.5	12.7	6.6	12.7
	Soccer	8.4	24.1	5.4	7.4
Cyclic	Kayaking	8.6	6.3	3.9	6.1
	Triathlon	5.6	7.1	4.1	4.7

Abbreviations: max = after test; 0 = before test

### Mean and individual hormonal response upon maximal workload

The differences between the mean concentration data of A, NA, DA, AGT, ET and C after and before of the applied test normalized with the basal concentration level of the corresponding neuroendocrine hormone and vasoconstrictor peptide in percentage are shown in Fig 1a. Among these normalized means, only C was negative in the case of controls. For A, a significant 7.7-fold increase in the normalized values were obtained for handball compared to kayaking (*p* = 0.035), while this percentage ratio for handball and triathlon was 5.7. Very similar ratios (7.5 *vs*. 5.7) were obtained for soccer and cyclic sports but the differences were not significant in this case (Fig 1a). Also, significant changes (*p* < 0.05) were observed for NA normalized concentration difference between each investigated ball game and cyclic sport activities (Fig 1a). Moreover, we established higher differences for the normalized concentration difference for handball and kayaking as well as handball and triathlon volunteers *p* being 0.00031 and 0.00034, respectively. Thus, the ratios calculated for the differences shown in Fig 1a for handball/kayaking, handball/triathlon, soccer/kayaking and soccer/triathlon were 5.4, 6.4, 3.8 and 4.6, respectively. For NA, there were also significant differences for control/handball, soccer/triathlon and soccer/kayak with p values being 0.047, 0.028 and 0.035, respectively (Fig 1a).

There were no significant changes in the concentration differences calculated in the similar way for the rest of the hormones and vasoconstrictor peptides.

We calculated the differences between the individual maximal workload and basal levels of hormonal, neurotransmitter and vasoconstrictor peptide concentration data normalized with the basal levels and expressed them in percentage for each volunteer (Fig 1b–1d). Then, we plotted those differences in increasing order of the calculated data for each sport activity. For catecholamines, higher *inter*individual variability has been found for ball game volunteers compared to volunteers of cyclic sports (Fig 1b). In the case of C, the responses of the triathletes seemed to be higher compared to all other volunteers (Fig 1d). The differences in the ET responses were always higher than zero for the soccer volunteers. Generally, positive differences in ET responses could be calculated in about 75% for the rest of the volunteers. We also indicated the *inter*individual variability of hormonal, neurotransmitter and vasoconstrictor peptide responses higher than the mean for at least three of the investigated compounds in the Fig 1b–1d with the ID code of the volunteers.

### Mean responses after and before the treadmill test normalized with maximal oxygen uptake

**Fig 2:**
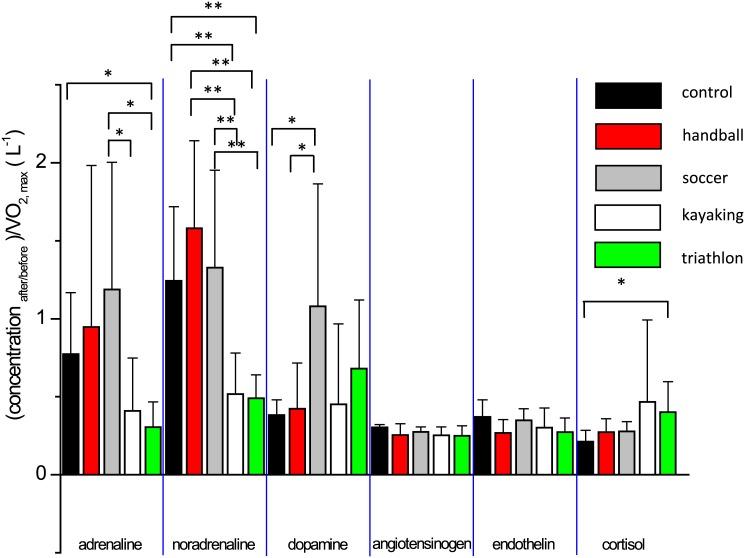
After—before concentration ratios of the investigated catecholamines, vasoconstrictor peptides and cortisol normalized with the maximal oxygen uptake. Significant differences (*p* < 0.05 and *p* < 0.01) are indicated with one or two asterisk(s), respectively; See text for exact *p* values.

We divided the hormonal neurotransmitter and vasoconstrictor peptide concentration levels after and before the treadmill test and normalized them by the corresponding VO_2max_ values for each volunteer and eventually, the mean ratios corresponding to each group were plotted (Fig 2). For A, we obtained significant increases of a factor of 2.9 and 3.9 in the case of soccer and cyclic sport volunteers, respectively, *p* being 0.0280 and 0.0314 by comparing the normalized means for soccer/kayaking and soccer/triathlon. Another significant difference was observable between control and triathlon volunteers (*p* = 0.0311). For NA, we could establish an even stronger significant difference roughly by a factor of 3 between each type of ball game volunteer mean data and that of each cyclic sport ones (*p* = 0.0000587 and *p* = 0.0000567 for handball/kayaking and handball/triathlon, respectively; *p* = 0.0028 and *p* = 0.0024 for soccer/kayaking and soccer/triathlon, respectively.). Similarly, the normalized after and before NA concentration ratios calculated for cyclic sport activity volunteers were significantly higher compared to the control group (*p* = 0.0021 and *p* = 0.0017 for kayaking/control and triathlon/control, respectively). For normalized similar DA ratios, *p* = 0.0463 and *p* = 0.0482 for handball/soccer and control/soccer, respectively. For the similar C ratios, there was only one significant difference, namely, between control and triathlon athletes, *p* being 0.04731.

## Discussion

To our knowledge, this is the first complex approach in terms of study design, selection of national elite athletes (handball and soccer as ball game *vs*. kayaker and triathletes as cyclic sport activity volunteers) and simultaneous recording of the response of several hormonal, neurotransmitter and vasoconstrictor compounds by performing a maximal exercise test for stress evaluation in the same training season. Due to the complex approach of the study we have highlighted the limitations and strengths of our present research (Table 5).

**Table 5:** All noted limitations and strengths.

Limitations	Strengths
General conclusion from a single type of stress test cannot be made and refer to a field training regimen.	Systematic in case of the observed stress hormones, neurotransmitter and vasoconstrictor concentrations.
Catecholamine concentrations are influenced by several factors—inter- and intraindividual comparism of hormonal neuroendocrine and vasoconstrictor peptide variations is challenging.	Standardize as much as possible the vita maxima tredmill test for all type of sports-suitable protocol.
Catecholamine values differ with gender and age—limitations of age and gender reduced the subject number.	Inclusion of the Hungarian elite athletes.
	All athletes were in the same training season (preparatory phase).
	Hormonal neurotransmitter and vasoconstrictor peptide concentration levels were normalized by the corresponding VO_2_max values
	Comparison of the different sport activities (cyclic versus ball game).

Since Zouhal et al. [5] reported that catecholamine values differ with gender and age (21 y *vs*. 34 y), volunteers having around 21 years of age were to be included into our study. Our primary selection criterion was to include first class athletes with several years of training experience. Due to limited number of national elite athletes in the aforementioned age range, only up to 12 volunteers could join the present study. Moreover, this selection criterion could not be met for handball players where the athletes had a slightly higher age as a whole national elite team could be monitored. Finally, we summed the individual workload values registered every minute during Bruce protocol, since these reflected better the differences in the workload of the volunteers (Table 5).

For the calculation of the fractionated mass data, we applied the method of Drinkwater and Ross [20], since this is the only method implemented for monitoring international level athletes in Hungary [21]. Differences in the height and weight of athletes are attributed to the different selection requirements of the certain sport activities. The absence of any significant difference in fractionated mass data of controls compared to the athletes can be explained with the recreational activities done by control volunteers. The lack of any significant difference in the muscle percentage of athletes is explained by the relatively similar training experience. The significant difference in the fat percentage of handball players compared to soccer ones may be attributed to their slightly increased age, body weight and physique. Whilst VO_2max_ of handball and triathlon volunteers had significantly higher values than the controls, the relative aerobic capacity expressed as VO_2max_ BW^-1^ was significantly higher only for triathletes because of having the highest endurance. This observation was supported by their cumulative workload, which was significantly the highest among almost all volunteers (Table 2).

The reason behind the different hormonal, neurotransmitter and vasoconstrictor peptide responses is the dissimilar requirements of the ball game *vs*. cyclic sport activities. Normalization of the hormonal, neurotransmitter and vasoconstrictor concentrations of each group by VO_2max_ is also recommendable as VO_2max_ is as an excellent indicator for endurance (Fig 2). The A and NA values obtained by us are similar to those reported in previous studies conducted on athletes. According to Chmura et al. [22], the maximum plasma NA and A concentrations during 20 min of exercise above the LAC threshold for soccer players achieved about 1 nmol L^-1^ and 6.0 nmol L^-1^, respectively. As it was expected according to the report of Jacob et al. [23], the highest change in LAC levels determined after and before the test was observed for controls, while the lowest for triathletes, the sport activity of the latter requiring endurance at most (Table 4).

The higher increase of A levels at maximum workload compared to the basal values for soccer and handball players (Figs 1a and 2) can be explained by taking into account that these highly competitive ball games require a combination of endurance, strength, power, sprinting and jumping skills where sudden efforts are needed for shorter time periods (i.e., during matches). Since kayaking and triathlon are considered more as cyclic sport activities requiring endurance, the lower response for A and NA levels can be understandable, in response to a maximal exercise test through Bruce protocol. As it was expected, the higher after and before NA concentration ratios for handball and soccer players are related to their increased motivation and drive to achieve the maximal workload [24]. According to Schulpis et al. [25], blood catecholamine levels of soccer players significantly increased in players postgame. In our study, the small changes in the C concentration ratios after and before the test were in good agreement with previous literature data. Bonifazi et al. [26] reported that handball players subjected to jumping sessions, plasma C levels varied between 334.4 ± 34.7 nmol L^-1^ and 416.3 ± 43.5 nmol L^-1^. In another study [27] evaluating testosterone and C levels in response to endurance training by running, the C concentration of endurance athletes ranged roughly between 1.0 and 4.0 ng L^-1^. These reported values were lower than those obtained in the present study but the type of training applied in our study differed considerably to that of aforementioned report. In turn, the C levels were 554.6 ± 95.3 nmol L^-1^ at the end of the training program and 612.2 ± 115.8 nmol L^-1^ in the middle as compared to the initial levels (442.9 ± 95.1 nmol L^-1^) [28]. Although these values reflecting the effect of a 12-week long training on soccer players were higher than those reported in the present study, the after and before C concentration ratios calculated were only slightly higher than those reported in this study (1.25 *vs*. 1.17, respectively). In our study, the C plasma concentration levels were even lower compared to the basal ones for controls. The fact that the C response compared to A is delayed can be an explanation for this phenomenon. Overtraining can also result in lower C responses [29], which was observed for some of the athletes involved in the present study (Fig 1d). It is also well known that the C levels increase for endurance athletes during performance. This is in accordance with our results (Fig 1a). Moreover, by normalizing C levels by VO_2max_, a significant change was observed between triathlon and control groups (Fig 2).

Endothelin plays an important role in the redistribution of blood flow exerting its effect predominantly locally. It was previously reported [11] that the exercise intensity of ventilatory threshold at 90% for 30 min increased the plasma concentration of ET 1.2-fold by exercise and that the changes in ET level were associated with the degree of exercise intensity. Legakis et al. [30] found difference in basal serum ET levels between 13 male professional football players and an equal number of sedentary or moderately physically active men of similar age and body mass index. The increased ET levels could be explained as being a consequence of a widening of the vascular bed resulting from increased muscle weight and size.

Maximal workload did not cause significant changes in AGT plasma levels in the present study maybe due to the fact that AGT does not take part in the circulation redistribution. This phenomenon suggests that long-term responses of AGT should be monitored.

Normalization of our data by VO_2max_ strengthened the *inter*group changes for the compounds monitored, since more statistically relevant and new *inter*group differences could be established for A and NA as well as DA and C. This outcome for DA and C is novel, since there is scarce information on the DA plasma levels for athletes subjected to different exercise programs and it is widely accepted that DA increases with exposure to various stressors. Taking into account the individual differences in the response levels of the studied six compounds normalized to the corresponding basal concentration, ball game athletes and control volunteers had more pronounced response levels in the case of almost every hormone except for C. Beside the group comparison, we found individual differences in the ET concentration responses after and before the test. Three handball pivots and three soccer players indicated in Fig 1c with ID codes 11, 12 and 13 as well as 19, 22 and 22, respectively, had higher ET concentration response than their sport group average (Fig 1c). Differences that we found in the ET individual concentration responses after and before the test raise the importance of studying this area more thoroughly among athletes [31].

## Conclusions

We found that a systematic comparison is needed for the complex evaluation of stress hormonal (A, NA, C), neurotransmitter (DA), as well as, vasoconstrictor peptides (AGT and ET) concentration variation in individual and team national elite athletes of cyclic and ball game sport activities, respectively, by executing a maximal exercise test through Bruce protocol on a treadmill. Normalization by VO_2max_ of the after and before concentration levels reflected better the differences in the response level of some hormonal neuroendocrine and vasoconstrictor compounds such as A, NA, DA and C.

In our study NA seemed to be a reasonable parameter to differentiate the ball games, cyclic sports and control group. Our results show that ball game athletes had the most pronounced NA response to acute stress (exercise test) followed by the control group and the lowest NA response occurred in the cyclic group. This tendency reveals the question of training adaptation in different sports. Higher NA secreting capacity during sport activity in ball games may be an advantageous adaptation mechanism to chronic training and the opposite can be assumable in cyclic sports based on our results.

Considering the limitation factors of our study this tendency that we have observed need to be further investigated to have direct conclusion on the exercise induced catecholamine plasma level changes in different sports.

## Supporting Information

S1 File Supporting information files. Fig A. Differences in the mean (± standard error, SE) (a) and individual concentration of catecholamines (b), vasoconstrictor peptides (c) and cortisol (d) between after and before test for the volunteers normalized with the basal concentration level of each corresponding neuroendocrine hormone and vasoconstrictor peptide. Fig B. After—before concentration ratios of the investigated catecholamines, vasoconstrictor peptides and cortisol normalized with the maximal oxygen uptake.
(DOCX)

## Abbreviations

A: adrenaline
AGT: angiotensinogen
ANOVA: analysis of variance
BW: body weight
C: cortisol
DA: dopamine
EDTA: ethylene diamine tetraacetic acid
ELISA: enzyme-linked immunosorbent assay
ET: endothelin
HR_max_: maximal heart rate
LAC: lactate
NA: noradrenaline
(R)SD: (relative) standard deviation
VO_2max_: maximal oxygen uptake
W_max_: maximal workload
