# Correction: Hormonal Neuroendocrine and Vasoconstrictor Peptide Responses of Ball Game and Cyclic Sport Elite Athletes by Treadmill Test

**DOI:** 10.1371/journal.pone.0153905

**Published:** 2016-04-13

**Authors:** Anna Protzner, Márta Szmodis, Anna Udvardy, Edit Bosnyák, Emese Trájer, Zsolt Komka, István Györe, Miklós Tóth

There is an error regarding equal contribution. Authors who are not first author Anna Protzner did not contribute equally to this work.
