# Supplementary material for: Hormonal Neuroendocrine and Vasoconstrictor Peptide Responses of Ball Game and Cyclic Sport Elite Athletes by Treadmill Test
Source: PLoS One. 2015 Dec 30;10(12):e0144691. doi: 10.1371/journal.pone.0144691 (PMC4696681; doi:10.1371/journal.pone.0144691)
Supplement: S1 File — Fig A. Differences in the mean (± standard error, SE) (a) and individual concentration of catecholamines (b), vasoconstrictor peptides (c) and cortisol (d) between after and before test for the volunteers normalized with the basal concentration level of each corresponding neuroendocrine hormone and vasoconstrictor peptide. Fig B. After—before concentration ratios of the investigated catecholamines, vasoconstrictor peptides and cortisol normalized with the maximal oxygen uptake. (DOCX) [file pone.0144691.s001.docx]

**Supporting Information**

**Legends to Supplementary Figures and Tables**

**Fig A**

**Differences in the mean (± standard error, SE) (a) and individual concentration of catecholamines (b), vasoconstrictor peptides (c) and cortisol (d) between after and before test for the volunteers normalized with the basal concentration level of each corresponding neuroendocrine hormone and vasoconstrictor peptide.**

Significant differences (*p* < 0.05 and *p* < 0.01) are indicated with one or two asterisk(s), respectively; See text for exact *p* values. Arabic numbers in Figure 1 b-d represent the volunteer identification code in the given group for which the difference in at least three hormone concentration levels after and before test was higher than the mean of the hormonal concentration difference.

**Fig B**

**After – before concentration ratios of the investigated catecholamines, vasoconstrictor peptides and cortisol normalized with the maximal oxygen uptake.** Significant differences (*p* < 0.05 and *p* < 0.01) are indicated with one or two asterisk(s), respectively; See text for exact *p* values.

**Supplementary Tables**

**Table A**

**Anthropometric data of the participants involved in the investigation (mean ± standard deviation, SD)**

^1^ according to Drinkwater and Ross (1980)

^a^ significant difference (*p* = 0.0326) compared to the control group; ^b^ significant difference (*p* = 0.0124) compared to the soccer athletes

**Table B**

**Exercise parameters after the treadmill test (mean values ± SD)**

Abbreviations: HR_max_ = maximal heart rate; LAC_max_ = maximal lactate level; VO_2max_ = maximal oxygen uptake; ^1^ significant difference for handball (*p* = 0.00874) and triathlon (*p* = 0.0496) compared to the control group; ^2^ significant difference for handball (*p* = 0.0249) and triathlon (*p* = 0.000194) compared to the control group, ^3^ significant difference *p* being 0.00628 and 0.00249 compared to the control group and handball athletes, respectively; ^a^ significant difference *p* being 0.00572 and 0.0183 compared to the soccer and kayaking athletes, respectively. ^*^Maximal lactate level was measured at the end of the exercise

**Table C**

**Concentration (mean ± SD, minimum – maximum range) of adrenaline (A), noradrenaline (NA), dopamine (DA), angiotensinogen (AGT), endothelin (ET) and cortisol (C) in athletes expressed as nmol L^-1^ before and after treadmill test.**

Significant intragroup changes (^1^, if p < 0.05 and ^2^, if p < 0.01). See text for exact p values.

**Table D**

**Adrenaline (A), noradrenaline (NA) and lactate (LAC) concentration ratios after and before the applied treadmill test**

Abbreviations: max = after test; 0 = before test

**Table E**

**All noted limitations and strengths**

**S1 Fig**

^*^

^*^

^**^

^*^

^**^

^*^

soccer

handball

triathlon

kayaking

control

**S2 Fig**

^**^

^**^

^**^

^**^

^**^

^*^

^*^

^*^

^*^

^*^

kayaking

soccer

triathlon

control

handball

**S1 Table**

| Sport activity | Volunteer | Nº volunteers (n) | Training experience (years) | Age ± SD (years) | Weight ± SD (kg) | Height ± SD (cm) | Fractionated mass^1^ |
| --- | --- | --- | --- | --- | --- | --- | --- |

|  |  |  |  |  |  |  | fat | muscle | bone | residual |
| --- | --- | --- | --- | --- | --- | --- | --- | --- | --- | --- |
|  |  |  |  |  |  |  | (% ± SD) | (% ± SD) | (% ± SD) | (% ± SD) |

|  | Control | 6 | - | 23.5 ± 1.9 | 76.9 ± 10 | 179.7 ± 2.7 | 13.9 ± 4.3 | 44.4 ± 3.3 | 16.7 ± 1.3 | 25.0 ± 1.7 |
| --- | --- | --- | --- | --- | --- | --- | --- | --- | --- | --- |
| Ball games | Handball | 12 | 15.6 ± 3.2 | 26.3 ± 3.5 | 101.7 ± 9.5 | 192.2 ± 6.9 | 14.4^b^ ± 3.1 | 44.2 ± 2.2 | 16.6 ± 1.1 | 24.8 ± 1.1 |
|  | Soccer | 8 | 10.2 ± 1.3 | 20.8 ± 2.3 | 79.4 ± 7.2 | 181.1 ± 7.5 | 9.02^a^ ± 1.5 | 48.0 ± 1.0 | 16.5 ± 0.9 | 26.5 ± 1.1 |
| Cyclic | Kayaking | 9 | 11.2 ± 0.8 | 19.5 ± 2.3 | 85.9 ± 8.1 | 184.1 ± 6.2 | 10.2 ± 0.8 | 47.9 ± 1.2 | 16.0 ± 0.5 | 25.9 ± 1.3 |
|  | Triathlon | 9 | 10.0 ± 2.1 | 20.4 ± 2.0 | 68.6 ± 12.0 | 177.6 ± 7.6 | 11.3 ± 2.2 | 44.3 ± 4.1 | 16.8 ±0.9 | 27.5 ± 2.2 |

**S2 Table**

| Sport activity | Volunteer | HR_max_ | VO_2max_ | Relative aerobic capacityVO_2max_ BW^-1^ | LAC_max_ | Cumulative workload(W) |
| --- | --- | --- | --- | --- | --- | --- |
|  |  | (beats min^-1^) | (mL min^-1^) | (mL min^-1^ kg^-1^) | (mmol L^-1^) |  |
|  | Control | 198 ± 8 | 3572 ± 438 | 47.0 ± 7.8 | 11.0 ± 1.7 | 2796 ± 885 |

| Ball games | Handball | 184 ± 12 | 4683^1^ ± 563 | 48.2 ± 9.1 | 10.7 ± 1.9 | 3886^2^ ± 591 |
| --- | --- | --- | --- | --- | --- | --- |
|  | Soccer | 195 ± 10 | 4209 ± 616 | 54.4 ± 5.6 | 12.4 ± 1.9 | 3440 ± 593 |
| Cyclic | Kayaking | 194 ± 8 | 4358 ± 652 | 56.4 ± 4.1 | 12.0 ± 0.9 | 3547 ± 313 |
|  | Triathlon | 198 ± 12 | 4541^1^ ± 788 | 66.1^3^± 13.9 | 12.3 ± 3.6 | 4690^2,a^ ± 887 |

**S3 Table**

| Sport activity | | Volunteer | A | | NA | | DA | | | |
| --- | --- | --- | --- | --- | --- | --- | --- | --- | --- | --- |
|  | |  | mean ± SD | | mean ±SD | | mean ±SD | | | |
|  | |  | min – max | | min – max | | min – max | | | |
| treadmill test |  | | before | after | before | after | before | | | after |
|  | | Control | 23.3 ± 8.9 | 56.3^1^ ± 20.9 | 172 ± 60 | 704^2^ ± 291 | 15.5 ±2.1 | | | 21.1 ± 6.2 |
|  | |  | 14.1 – 38.6 | 31.7 – 93.0 | 91.4 – 271 | 268 – 1225 | 11.4 – 18.1 | | | 14.5 – 33.1 |
| Ball games | | Handball | 9.9 – 53.3 | 136^2^ ± 53 | 267 ± 88 | 1719^2^ ± 607 | 25.7 ± 12.6 | | | 43.7 ± 15.3 |
|  |  |  | 40.1 ± 21.7 | 59.2 – 229 | 176 – 509 | 618 – 2465 | <0.15 – 46.0 | | | 24.9 – 81.5 |
|  | | Soccer | 29.1 – 214 | 299^2^ ± 169 | 457 ± 158 | 2204^2^ ± 413 | 34.2 ± 26.7 | | | 74.6^1^ ± 27.4 |
|  |  |  | 84.8 ± 61.4 | 153 – 626 | 193 – 637 | 1551 – 2843 | 8.3 – 83.5 | | | 26.9 – 105 |
| Cyclic | | Kayaking | 27.6 – 87.6 | 76 ± 34 | 218 ± 79 | 461^2^ ± 230 | 27.2 ± 20.2 | | | 32.6 ± 17.2 |
|  |  |  | 56.5 ± 21.6 | 37.2 – 135 | 134 – 362 | 231 – 955 | 4.0 – 57.2 | | | 16.7 – 68.8 |
|  | | Triathlon | 32.8 – 82.8 | 87.7^2^ ± 25.5 | 234 ± 83 | 414 ±178 | 12.3 ± 6.1 | | | 27.6^2^ ± 10.6 |
|  |  |  | 57.5 ± 18.6 | 55.5 – 127 | 152 – 432 | 90.7 – 730 | 4.8 – 23.1 | | | 12.2 – 43.0 |
|  | |  | AGT | | ET | | C | | | |
|  | |  | mean ±SD | | mean ±SD | | mean ±SD | | | |
|  | |  | min – max | | min – max | | min – max | | | |
| treadmill test |  | | before | after | before | after | before | after | | |
|  | | Control | 2.7 ± 0.4 | 2.9 ± 0.4 | 6.4 ± 1.3 | 8.2^1^ ± 1.9 | 159 ± 38 | | 119 ±50 | |
|  | |  | 2.2 – 3.5 | 2.4 – 3.5 | 5.1 – 9.3 | 5.3 – 11.5 | 98.2 – 216 | | 55.8 – 200 | |
| Ball games | | Handball | 3.2 ± 0.8 | 3.6 ± 1.0 | 7.4 ± 2.6 | 8.8^1^ ± 3.0 | 130 ± 45 | | 155^1^ ± 46 | |
|  |  |  | 2.0 – 4.7 | 2.1 – 4.6 | 4.8 – 15.1 | 4.2 – 15.9 | 83.4 – 222 | | 104 – 250 | |
|  | | Soccer | 3.0 ± 0.6 | 3.5^2^ ± 0.6 | 10.9 ± 1.5 | 16.1^2^ ± 1.0 | 141 ± 46 | | 165 ±71 | |
|  |  |  | 2.0 – 3.9 | 2.4 – 4.4 | 9.5 – 13.1 | 14.6 – 17.2 | 73.7 – 223 | | 91.8 – 316 | |
| Cyclic | | Kayaking | 3.1 ± 0.6 | 3.3 ± 0.8 | 10.5 ± 2.9 | 13.8 ± 6.6 | 164 ± 103 | | 188 ± 99 | |
|  |  |  | 2.2 – 4.0 | 2.4 – 4.7 | 6.2 – 13.4 | 6.0 – 26.0 | 3.7 – 333 | | 23.0 – 381 | |
|  | | Triathlon | 3.9 ± 0.9 | 4.2 ± 1.0 | 11.8 ± 3.9 | 12.6 ±3.3 | 119 ± 51 | | 211^2^ ± 40 | |
|  |  |  | 2.5 – 5.3 | 2.9 – 5.5 | 6.3 – 16.4 | 7.6 – 19.2 | 51.9 – 205 | | 163 – 293 | |

**S4 Table**

| Sport activity | Volunteer | LAC_max_/LAC_0_ | A_max_/LAC_max_ | NA_0_/A_0_ | NA_max_/A_max_ |
| --- | --- | --- | --- | --- | --- |
|  | Control | 9.2 | 5.1 | 7.4 | 12.5 |
| Ball games | Handball | 6.5 | 12.7 | 6.6 | 12.7 |
|  | Soccer | 8.4 | 24.1 | 5.4 | 7.4 |
| Cyclic | Kayaking | 8.6 | 6.3 | 3.9 | 6.1 |
|  | Triathlon | 5.6 | 7.1 | 4.1 | 4.7 |

**S5 Table**

| **Limitations** | **Strengths** |
| --- | --- |
| General conclusion from a single type of stress test cannot be made and refer to a field training regimen. | Systematic in case of the observed stress hormones, neurotransmitter and vasoconstrictor concentrations. |
| Catecholamine concentrations are influenced by several factors – inter- and intraindividual  comparism of hormonal neuroendocrine and vasoconstrictor peptide variations  is challenging. | Standardize as much as possible the vita maxima tredmill test for all type of sports-suitable protocol. |
| Catecholamine values differ with gender and age - limitations of age and gender reduced the subject number. | Inclusion of the Hungarian elite athletes. |
|  | All athletes were in the same training season (preparatory phase). |
|  | Hormonal neurotransmitter and vasoconstrictor peptide concentration levels were normalized by the corresponding VO_2_max values |
|  | Comparison of the different sport activities (cyclic versus ball game). |
